# APPROACH: Sensitive Detection of Exosomal Biomarkers by Aptamer-Mediated Proximity Ligation Assay and Time-Resolved Förster Resonance Energy Transfer

**DOI:** 10.3390/bios14050233

**Published:** 2024-05-08

**Authors:** Ying Li, Meiqi Qian, Yongpeng Liu, Xue Qiu

**Affiliations:** 1 Laboratory for Marine Drugs and Bioproducts, Qingdao National Laboratory for Marine Science and Technology, Key Laboratory of Marine Drug, Ministry of Education, School of Medicine and Pharmacy, Ocean University of China, Qingdao 266003, China; liying9239@stu.ouc.edu.cn (Y.L.); qmq@stu.ouc.edu.cn (M.Q.); 2 BGI Research, Shenzhen 518083, China; liuyongpeng@genomics.cn

**Keywords:** exosomal biomarkers, aptamer, proximity ligation assay, rolling circle amplification, time-resolved Förster resonance energy transfer

## Abstract

Exosomal biomarker detection holds great importance in the field of in vitro diagnostics, offering a non-invasive and highly sensitive approach for early disease detection and personalized treatment. Here, we proposed an “APPROACH” strategy, combining aptamer-mediated proximity ligation assay (PLA) with rolling circle amplification (RCA) and time-resolved Förster resonance energy transfer (TR-FRET) for the sensitive and semi-homogenous detection of exosomal biomarkers. PLA probes consisted of a cholesterol-conjugated oligonucleotide, which anchored to the membrane of an exosome, and a specific aptamer oligonucleotide that recognized a target protein of the exosome; the proximal binding of pairs of PLA probes to the same exosome positioned the oligonucleotides in the vicinity of each other, guiding the hybridization and ligation of two subsequently added backbone and connector oligonucleotides to form a circular DNA molecule. Circular DNA formed from PLA underwent rolling circle amplification (RCA) for signal amplification, and the resulting RCA products were subsequently quantified by TR-FRET. The limits of detection provided by APPROACH for the exosomal biomarkers CD63, PD-L1, and HER2 were 0.46 ng∙μL^−1^, 0.77 ng∙μL^−1^, and 1.1 ng∙μL^−1^, respectively, demonstrating excellent analytical performance with high sensitivity and quantification accuracy. Furthermore, the strategy afforded sensitive detection of exosomal CD63 with a LOD of 1.56 ng∙μL^−1^ in complex biological matrices, which underscored its anti-interference capability and potential for in vitro detection. The proposed strategy demonstrates wide-ranging applicability in quantifying diverse exosomal biomarkers while exhibiting robust analytical characteristics, including high sensitivity and accuracy.

## 1. Introduction

Exosomes are nanoscale extracellular vesicles released by various cells under physiological and pathological conditions [1]. They facilitate intercellular communication by transporting diverse molecular cargoes, which can regulate recipient cell functions and contribute to disease progression [2]. Owing to the stability afforded by their lipid bilayer encapsulation and accessibility through readily accessible body fluids, exosomes exhibit the potentiality of assisting with disease diagnostic and therapeutic assessment [3,4]. Tumor-derived exosomes carry common vesicle transmembrane proteins (CD9, CD63, and CD81) as well as cancer-associated membrane proteins (EpCAM, EGFR, and PD-L1) [2,5]. Recently, researchers have developed novel analytical assays for exosomal biomarkers based on fluorescence [6] and electrochemical detection [7], Raman spectroscopy [8], and mass spectrometry [9]. These techniques could achieve highly sensitive and specific quantification of exosomal biomarkers; however, they also come with certain limitations. Electrochemical methods offer advantages such as simplicity, low cost, and rapid detection, making them suitable for point-of-care applications, but they are also prone to interference from additional electroactive substances present within complex biological matrices, potentially compromising the precision of the tests [10]. Methods based on Raman spectroscopy and mass spectrometry provide valuable information on molecular composition and can achieve high specificity, though they often require specialized expertise and time-consuming sample preparation, limiting the widespread applicability for point-of-care diagnostics and personalized treatment [11]. Therefore, more simplified and sensitive exosomal biomarker quantification techniques are needed and could pave the way towards the clinical utility of liquid biopsies with exosomes.

Aptamers—short single-stranded DNAs or RNAs—have gained significant attention in recent years as promising molecular recognition elements for various biomedical applications [12,13]. Thanks to an intensive investigation of the systematic evolution of ligands through the exponential enrichment (SELEX) technique over last 20 years [14], new variations of SELEX such as bead-[15], capillary electrophoresis-[16], and cell-based SELEX [17] have emerged, markedly enhancing the affinity and specificity of the screened aptamers. Up to now, thousands of protein-binding aptamers [18] and several hundred small molecule-binding aptamers [19] have been reported, and their affinities to the targets can reach sub-nM [20]. With their high specificity, affinity, and accessibility, aptamers are very well-suited for the detection and analysis of biomarkers [21,22] and have proved to be powerful alternatives to antibodies. Among the emerging techniques utilizing aptamers, the combination of aptamers with proximity ligation analysis (PLA) has become known as a powerful approach to biomarker detection [23]. PLA, pioneered by Fredriksson et al. in 2002 [24], integrates immunoassays with nucleic acid-based amplification, thereby enhancing analytical sensitivity. It yields signals solely upon close proximity to two PLA probes, conferring exceptional specificity. Luo et al. demonstrated that employing PLA effectively reduces background signals caused by exosome disruption or the release of free proteins from cells [6].

PLA is commonly coupled with efficient nucleic acid amplifications [25], such as quantitative real-time polymerase chain reaction (qRT-PCR), rolling circle amplification (RCA), or catalytic hairpin assembly (CHA), for quantitative analysis by fluorescence [26,27]. Traditional fluorescence-based techniques for quantifying amplification products suffer from interference by autofluorescence in complex biological samples, and this issue can be greatly improved by introducing Förster resonance energy transfer (FRET) and time-resolved FRET (TR-FRET). FRET occurs between two different fluorophores when the emission spectrum of the donor fluorophore partly overlaps with the absorption spectrum of the acceptor, and it can only be observed when the distance between the two fluorophores is appropriately close (typically less than 10 nm) [28,29]. Upon exciting the donor, the emitted fluorescence from the acceptor can be observed [30]. Due to the inherent characteristics of FRET, unbound donor and acceptor molecules in solutions do not contribute to the FRET signal [31], thereby facilitating the implementation of homogeneous detection with high efficiency. TR-FRET combines the homogeneous detection capabilities of FRET with the low-background advantages of time-resolved fluorescence measurements, which overcomes the limitations of traditional fluorescence-based techniques [32]. TR-FRET frequently utilizes lanthanide complexes as donors owing to their long fluorescence lifetimes [33,34], which allows time-gated fluorescence intensity measurements [35]. Lanthanide (e.g., Tb or Eu) complexes are intriguing FRET donors due to their notably high photoluminescence (PL) quantum yields, exceptionally prolonged PL lifetimes, and multiple emission bands spanning a wide spectral range [36]. By selecting the appropriate temporal window, background interference can be eliminated [37]. This enhances assay sensitivity and resistance to interference for quantifying biomarkers even at low concentrations in complex matrices like biofluids [38]. Lanthanide-based TR-FRET has resulted in significant advances in high-throughput drug screening [39] and point-of-care in vitro diagnostics [40], with high sensitivity and selectivity. Compared to other fluorescence techniques, TR-FRET offers advantages including multiplexing capability, reduced matrix effects, and attomolar detection limits [41], rendering it well-suited for quantitative exosomal biomarker analysis.

Herein, we propose an “APPROACH” strategy combining aptamer-mediated PLA with RCA and TR-FRET for the sensitive and semi-homogenous detection of exosomal biomarkers. The semi-homogenous detection was performed by applying aldehyde/ketone sulfonic acid microspheres for the enrichment of exosomes while removing unbound PLA probes through a single separation step. The usage of aptamers simply converted the abundance of protein biomarkers into nucleic acid signals and facilitated the following signal amplification. PLA and RCA were then employed to shield against interference from free proteins and enable signal amplification. Incorporating TR-FRET detection allowed direct fluorescence quantification without separation and avoided background interference. We chemically modified a recently developed CoraFluor [36] with a like-NHS ester for oligonucleotide labeling and applied it as the FRET donor. CD63, programmed cell death ligand 1 (PD-L1), and human epidermal growth factor receptor 2 (HER2) were selected as model exosomal biomarkers to test the performance of the newly developed strategy.

## 2. Materials and Methods

### 2.1. Chemicals and Reagents

All oligonucleotides were purchased from Sangon Biotech (Shanghai, China) and purified by high-performance liquid chromatography (HPLC). The Cy5-NHS was obtained from the Lumiprobe Corporation (Cockeysville, MD, USA). Bovine serum albumin (Cat #. A8010) was purchased from Beijing Solarbio Science & Technology Co., Ltd. (Beijing, China). IgGs against CD63 (Cat #. ab134045), CD9 (Cat #. ab263019), and CD81 (Cat #. ab109201) were provided by Abcam Trading Co., Ltd. (Shanghai, China). A 7 kDa Zeba desalting column (Cat #. 89883), T4 DNA ligase (Cat #. EL0011), and phi29 DNA polymerase (Cat #. EP0091) were obtained from Thermo Fisher Scientific (Waltham, MA, USA). The BCA kit (Cat #. P0010) was provided by Beyotime Biotechnology Inc. (Shanghai, China). Aldehyde/sulfate latex microspheres (4%, 4μm) were purchased from Beijing Beiao Tai Biological Technology Co., Ltd. (Beijing, China). DMF (Cat #. 648531), N, N-diisopropylethylamine (Cat #. 387649), and TbCl_3_·6H_2_O (Cat #. 204560) were provided by Sigma (St. Louis, MO, USA). dNTPs (Cat #. N0447S) were obtained from New England Biolabs (Ipswich, MA, USA).

### 2.2. Cell Lines and Cell Culture

A human neuroblastoma cell line (SH-SY5Y) and a human non-small cell lung cancer cell line (A549) were obtained from the Stem Cell Bank of the Chinese Academy of Sciences (Beijing, China). SH-SY5Y and A549 cells were cultured in complete media prepared with high-glucose DMEM and RPMI 1640, respectively. The complete media contained 10% fetal bovine serum (ShuangRu Biotech, Suzhou, China) and 1% penicillin–streptomycin mixture (Solarbio Science & Technology Co., Ltd., Beijing, China). To remove exosomes derived from fetal bovine serum, the serum was centrifuged at 100,000× *g*, 4 °C for 16 h, and the resulting exosome-free complete medium was further prepared. The cells were cultured in a 100% humidified atmosphere of 5% CO_2_ at 37 °C.

### 2.3. Exosome Isolation and Characterization

Exosomes were isolated from the cell culture supernatant using ultracentrifugation as described in the literature [42,43]. Upon reaching 80% confluence, cells were washed with PBS to remove dead cells and residual media, then cultured in exosome-free complete media for 48 h. The cell culture supernatant was collected, and the collection process for exosomes was as follows: exosomes were isolated by differential centrifugation at 4 °C for 10 min (first at 300× *g*, subsequently at 2000× *g*, and next at 10,000× *g*). Finally, the pellet containing exosomes was obtained by centrifuging at 100,000× *g*, 4 °C for 70 min. The isolated exosomes were resuspended in PBS and the above washing steps were then repeated. Finally, the purified exosomes were resuspended in 500 μL of PBS and stored in aliquots at −80 °C for subsequent experiments.

For the characterization of exosomes, transmission electron microscopy (TEM) imaging was performed using the Talos L120C (Thermo Fisher Scientific, Waltham, MA, USA) at 120 kV. The concentrations and size distributions of the exosomes were measured using the Flow NanoAnalyzer (Nanaofcm, Xiamen, China). The total exosomal proteins were determined by the BCA kit, and the expression of characteristic proteins (CD63, CD9, CD81) was analyzed by Western blot analysis.

### 2.4. Synthesis of CoraFluor-Like-NHS

The Cora-H ligand was synthesized following a previously reported method [36]. For the preparation of Cora-like-NHS, TNTU (54.7 mg, 0.15 mmol, 1.5 eq) and N, N-diisopropylethylamine (19.5 mg, 0.15 mmol, 1.5 eq) were mixed with Cora-H ligand (116.3 mg, 0.10 mmol, 1.0 eq) dissolved in DMF and the reaction was allowed to proceed at room temperature for 2 h. Subsequently, TbCl_3_∙6H_2_O (37.4 mg) was added to the above mixture and stirred for 2 h at room temperature to yield a well-prepared CoraFluor-like-NHS. The solution was stored and protected from light at −20 °C for the following labeling procedures. A high-resolution mass spectrometer (Thermo Fisher Scientific, LTQ ORBITRAP XL, Waltham, MA, USA) was used with electrospray ionization (ESI) to analyze molecular weight. C_67_H_81_N_13_O_16_Tb, [M-2H]^−^ calcd: 1480.5021, found: 1480.5031 (Figure S3).

### 2.5. Fluorescent Labeling of Oligonucleotides

Amino-modified oligonucleotides were conjugated to Tb or Cy5 via NHS ester. A 12-fold molar excess of CoraFluor-like-NHS or Cy5-NHS was left to react with amino-modified oligonucleotides in carbonate buffer (100 mM NaHCO_3_, pH = 9.0) overnight at 4 °C. The conjugates were purified with HEPES Buffer (100 mM, pH 7.4) by a Zeba spin desalting column (7 kDa MWCO). The concentrations of Tb and Cy5 were determined by measuring the absorbance at 340 nm and 650 nm, respectively, with an ultraviolet spectrophotometer (HITACHI U3900, Tokyo, Japan). Oligonucleotides were quantified by absorbance measurements at 260 nm. Conjugation ratios were determined by linear combination of the respective absorbance values of Tb (or Cy5) and oligonucleotides within the dye-oligonucleotides conjugates. Emission spectra were acquired using a photoluminescence spectrometer equipped with a microplate reader (Edinburgh Instruments, FLS 1000, Edinburgh, UK) in HEPES Buffer. The Tb probe was excited at 340 nm with a bandwidth of 5 nm, while Cy5-oligonucleotides were excited at 590 nm with a bandwidth of 5 nm. Emission spectra were collected, ranging from 450–700 nm and 600–800 nm, respectively (Figure S1).

### 2.6. Flow Cytometry Analysis of the Binding Affinities between Cho Primer/Apt-CD63 and Exosomes

SH-SY5Y exosomes were deposited on aldehyde/ketone microspheres in order to be detectable in flow cytometry. First, 20 µL of exosomes was incubated with 2 μL of aldehyde/ketone microspheres for 15 min, followed by adding 250 μL 1 × PBS for another 2 h. Then, 200 μL blocking buffer (1 × PBS, 1M glycine and 2% BSA (*w*/*v*), Buffer-1) was added and incubated for 1 h. Afterwards, the exosome-coated microspheres were washed twice with 1 × PBS by centrifugation (8000 rpm, 3 min). Next, the purified exosome-coated microspheres were incubated with 200 nM Cho primer-Cy5 or Apt-CD63-Cy5 in binding buffer (1 × PBS, 5 mM MgCl_2_, and 0.2% BSA (*w*/*v*), Buffer-2) at 37 °C for 1 h. After two rounds of washing, the fluorescence intensity of the exosome-coated microspheres was analyzed with a flow cytometer (ACEA, NovoCyte 3080, San Diego, CA, USA) by capturing 50,000 events. The Cy5 fluorescence signal was collected in the APC channel with a 638 nm laser and a 660 ± 10 nm band-pass filter.

### 2.7. PLA and RCA Reactions

Microspheres coated with various concentrations of exosomes were incubated with 10 nM Cho primer and aptamer (Apt-CD63 for SH-SY5Y cell-derived exosomes, Apt-PD-L1 and Apt-HER2 for A549 cell-derived exosomes) in 100 μL Buffer-2 at 37 °C for 1 h. Free PLA probes were removed by centrifugation at 8000 rpm for 5 min. The exosome-coated microspheres were mixed with 10 μL T4 DNA ligase reaction buffer (40 mM Tris-HCl, 10 mM MgCl_2_, 10 mM DTT, 0.5 mM ATP, pH 7.8, Buffer-3) comprising 50 nM backbone, 50 nM connector, and 5 U T4 DNA ligase, to initiate the PLA reaction and generate a circular RCA template. Then 5 μL phi29 DNA polymerase reaction buffer (33 mM Tris-acetate, 10 mM Mg-acetate, 66 mM K-acetate, 1% (*v*/*v*) Tween 20, 1 mM DTT, pH 7.9, Buffer-4), which contained 5U phi29 DNA polymerase and 1.5 mM dNTPs mixture, was added to initiate the RCA reaction at 37 °C for 2 h.

### 2.8. Validation of RCA Products by Confocal Microscopy

The Cy5 probe (125 nM) was added to the SH-SY5Y exosome-coated microspheres with RCA products and incubated at 37 °C for 2 h in 5 μL hybridization buffer (20 mM Tris-Cl, 500 mM NaCl, pH 8.0, Buffer-5). Finally, the reaction mixture was centrifuged at 8000 rpm for 5 min to remove the supernatant, then resuspended in 10 μL PBS. The suspension was dropped onto a glass slide, covered with a coverslip, and sealed with glass adhesive. The imaging was collected with a 100× oil immersion objective in a confocal microscope (Leica, SP8 STED 3X, Hessen, Germany) without applying the STED super-resolved mode. Cy5 fluorescence was excited by choosing the light of 649 nm from a white light laser (spectrum ranging from 470 nm to 670 nm) and collected from 660 nm to 755 nm (PMT detector, 650 V).

### 2.9. Verification of RCA Products by Flow Cytometry

The resultant SH-SY5Y exosome-coated microspheres, with RCA products binding to the Cy5 probe, were resuspended in 500 μL PBS and then analyzed for fluorescence intensity by capturing 50,000 events with a flow cytometer. The Cy5 fluorescence signal was collected in the APC channel with a 638 nm laser and a 660 ± 10 nm band-pass filter.

### 2.10. Exosomal Biomarker Analysis by APPROACH Strategy

Tb probe and Cy5 probe (250 nM) prepared in 5 μL Buffer-5 were added to SH-SY5Y exosome-coated microspheres (or A549 exosome-coated microspheres) with RCA products and incubated in a thermal cycler with a temperature control program (65 °C for 10 min → decreased from 65 °C to 22 °C with a 2 °C min^−1^ speed → 22 °C for 10 min). Fluorescence decay curves were acquired directly through the photoluminescence spectrometer equipped with a microplate reader, using nitrogen laser (MNL 100 Nitrogen Laser, LTB Lasertechnik Berlin GmbH, Berlin, Germany) excitation (337.1 nm, 20 Hz, 100 pulses). Tb was detected at 552 nm with a bandwidth of 10 nm, while Cy5 was detected at 665 nm with a bandwidth of 20 nm. Time-gated (0.1–0.5 ms) PL intensity measurements were taken in black 384-well microtiter plates using a multifunctional microplate reader (TECAN Spark, Männedorf, Switzerland) using optical band-pass filters with 340 ± 20 nm for excitation, 552 ± 10 nm for the Tb detection channel, and 665 ± 8 nm for the Cy5 detection channel.

## 3. Results and Discussion

### 3.1. Principles of APPROACH Strategy for Exosome Detection (Figure 1)

Aldehyde/sulfate latex microspheres were firstly applied in the APPROACH strategy to enable the enrichment of exosomes, and then the exosome-coated microspheres were treated with PLA probes, which consist of a specific aptamer (including a target-specific sequence and the complementary sequence of the backbone/connector) that recognizes exosomal biomarkers and a cholesterol-modified primer (Cho primer) inserted into lipid membranes. Subsequently, the unbound PLA probes were removed by centrifugation. The following PLA, RCA, and TR-FRET processes were executed during a homogeneous phase. Ideally, a complete homogenous assay is preferred for simple biosensing; however, within the PLA process, the absence of separation led to the non-specific binding of non-negligible free PLA probes to the backbone and connector, resulting in indistinguishable RCA products that interfered with the accurate quantification of specific exosomal biomarkers, and in this situation, the introduction of a single separation step with microspheres became necessary and performance-effective.

**Figure 1:**
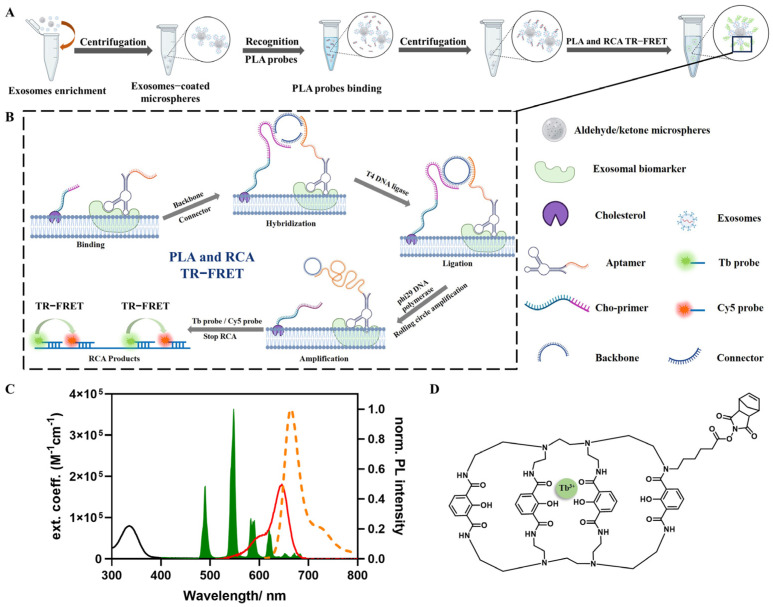
The principles of the APPROACH strategy for exosomal biomarker detection. (**A**) The workflow of the APPROACH strategy. (**B**) Schematic details of the PLA, RCA, and TR−FRET steps. (**C**) The absorption spectra of Tb (black) and Cy5 (red), and the emission spectra of Tb (green) and Cy5 (orange). (**D**) The chemical structures of CoraFluor−like−NHS (Tb).

The following procedure of the APPROACH strategy with the PLA, RCA, and TR-FRET steps is depicted in detail in Figure 1B. The PLA probes were strategically anchored to adjacent membrane proteins and cholesterol, converting the presence of proteins into nucleic acid signals amenable to amplification. The spatial arrangement guided the free terminals of the PLA probes into proximity, facilitating the formation of a circular DNA strand in the presence of backbone and connector oligonucleotides. Subsequently, the circular DNA acted as a template, and the aptamer probes (Apt-CD63, Apt-PD-L1, or Apt-HER2) served as a primer for localized RCA, while the Cho primer was unwound from the DNA circle. After the generation of RCA products, CoraFluor-like-NHS (Tb) and Cy5 (refer to Figure 1C for absorption and emission spectra) labeled the oligonucleotides hybridized to RCA products, with Tb and Cy5 being separated at a distance of 15 base pairs, and the detection was based on time-gated FRET from Tb to Cy5. The chemical structure of CoraFluor-like-NHS is depicted in Figure 1D. The time-gated intensities of the Tb donor and Cy5 acceptor were obtained by either counting the photons within the 0.1–2 ms time window of the fluorescence decay curves, or via the TRF mode of a multifunctional microplate reader. The FRET ratio was calculated by dividing the intensity of the Cy5 acceptor ($I_{Cy5}^{TG}$) by that of the Tb donor ($I_{Tb}^{TG}$) (Equation (1)). The relative FRET ratio represents the relative change in the FRET ratio compared to that in the absence of a target (Equation (2)).

(1) $$FRET\;Ratio=\frac{I_{Cy5}^{TG}}{I_{Tb}^{TG}}$$

(2) $$Relative\;FRET\;Ratio=\frac{FRET\;Ratio}{FRET\;Ratio(0)}$$

The spatially resolved nature of TR-FRET eliminates the requirement of additional separation steps for the unbound Tb probe and Cy5 probe. The sequences and modifications of all oligonucleotides used are summarized in Table S1 in the supporting information.

### 3.2. Characterization of Exosomes

**Figure 2:**
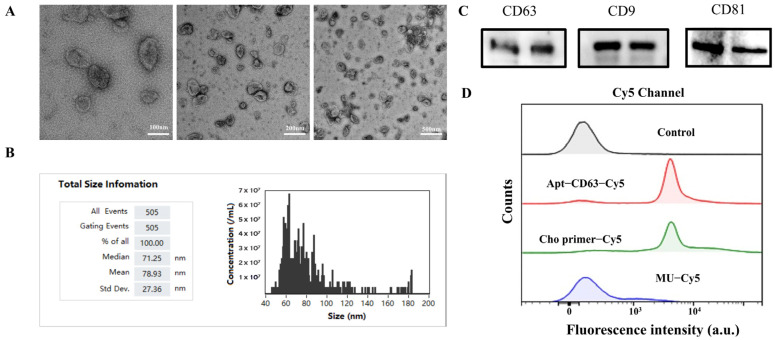
(**A**) The TEM images of the exosomes. (**B**) The sizes of the exosomes, measured with a flow nanoanalyzer. (**C**) The typical proteins present on exosomes, shown by Western blot. (**D**) The flow cytometry analysis of the capability of Cho primer in incorporating into exosomes and the binding affinity of Apt−CD63 to CD63 proteins.

The exosomes extracted from SH-SY5Y cells were characterized using TEM and a flow nanoanalyzer for morphological observation, and Western blot for specific protein validation. The TEM images, shown in Figure 2A, provided evidence that the extracted exosomes from SH-SY5Y cells presented typical cup-shaped double-layer membrane structures. The results obtained from the flow nanoanalyzer, shown in Figure 2B, indicated that the diameters of the isolated exosomes ranged from 70 nm to 150 nm, aligning with most previously reported findings [44]. We subsequently conducted an analysis of three marker proteins (CD63, CD9, and CD81) expressed on the surfaces of the isolated exosomes through Western blot (Figure 2C), and the results provided additional validation regarding the purity and integrity of the exosomes.

Considering that the binding affinities of the PLA probes are essential for proximity efficiency, we labeled the PLA probes directly with Cy5 to assess their binding affinities to SH-SY5Y cell-derived exosomes through flow cytometry. Next, 4 μm of aldehyde/sulfate latex microspheres was employed to facilitate the enrichment of the sub-200 nm exosomes and fulfil the requisite conditions for flow cytometric analysis. After centrifugation, the microspheres were washed twice with PBS to mitigate background fluorescence from unbound probes. As shown in Figure 2D, co-incubation of the exosome-coated microspheres with either Cy5-labeled Apt-CD63 or Cy5-labeled Cho primer introduced a 10^3^-fold increase in fluorescence intensity in the Cy5 channel compared to that of the control group and a random sequence (MU-Cy5), clearly demonstrating the high binding affinities of both probes to exosomes. Notably, the Cho primer exhibited nearly identical Cy5 fluorescence intensity compared to Apt-CD63, which was beneficial for the assembly of the following APPROACH strategy.

### 3.3. Feasibility and Visualization Analyses of the APPROACH Strategy for Exosomal Biomarker Detection

**Figure 3:**
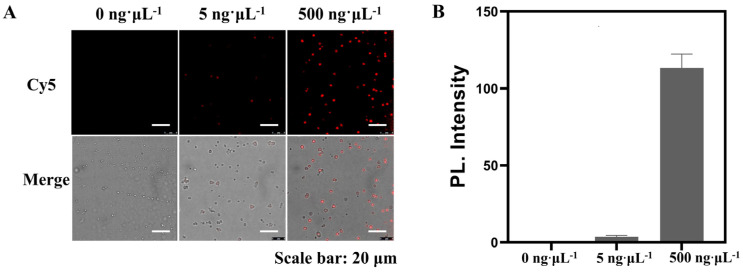
(**A**) An image of SH−SY5Y exosomal CD63 detected through the APPROACH strategy. The images were excited with a 649 nm laser and emissions from 660−755 nm were collected. (**B**) The quantitative fluorescence intensity analysis by ImageJ. The error represents the variability in signal intensity across different exosome−coated microspheres. Scale bars: 20 μm.

After extracting the qualified exosomes, we continued to investigate whether the proposed APPROACH strategy could be used for accurate SH-SY5Y exosome identification. Microspheres with different amounts of exosomes (0 ng∙μL^−1^, 5 ng∙μL^−1^, 500 ng∙μL^−1^) extracted from SH-SY5Y were mixed with PLA probes (Cho primer and Apt-CD63 for exosomal CD63 detection), and after the centrifugation step to get rid of the free PLA probes, backbone and connector oligonucleotides and T4 DNA ligase were added to obtain the circular DNA. The circular DNA was then used as the template to perform RCA. Confocal microscopy and flow cytometry were employed to characterize the RCA products with a Cy5 probe. As illustrated in Figure 3A, there was no fluorescence signal observed in the Cy5 channel for the group without exosomes, indicating that the microspheres would not produce non-specific signals of Cy5 probes. Concurrently, as exosome concentrations were gradually increased, the fluorescence signals exhibited a fluctuating pattern. Quantitative analysis of the fluorescence intensity is shown in Figure 3B. When the concentration of exosomes rose to 500 ng∙μL^−1^, a significantly enhanced fluorescence intensity was noted in the Cy5 channel compared to that of the control sample. It was reported that direct stochastic optical reconstruction microscopy (dSTORM) can be applied for the rapid detection of exosomes down to 20–30 nm in size, with high sensitivity and low variability [45]. Both imaging strategies could be performed on wide-field microscopes, with distinct advantages. dSTORM can achieve single-molecule localization and allows for super-resolution imaging without fluorescence signal amplification, while microsphere-agminated and aptamer-mediated PLA and RCA products can be observed as bright spots following the direct excitation of a continuous light source and do not require repetitive activation, localization, and deactivation with different light sources, as is necessary for dSTORM. Both strategies could be applied as alternatives for sensitive exosome imaging.

The flow cytometry results (Figure S2) also revealed significantly stronger fluorescence signals in PLA-RCA samples compared to those of exosomes that did not undergo the PLA process and RCA amplification (approximately 10^2^-fold enhancement), highlighting the capacity of the sensing strategy for detecting low-abundance exosomal biomarkers. The results obtained from confocal microscopy and flow cytometry collectively confirmed the successful establishment of an aptamer-mediated PLA and RCA strategy for sensitively transforming the information of exosomal biomarkers into amplified and detectable fluorescence intensities.

### 3.4. Optimization of Experimental Conditions

**Figure 4:**
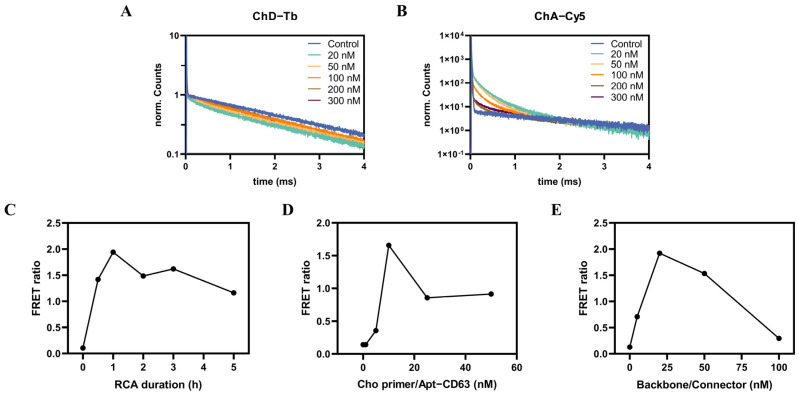
The optimization of the experimental conditions. The normalized fluorescence decay curves of the donor (**A**) and acceptor channels (**B**) corresponding to various concentrations (ranging from 20 nM to 300 nM) of Tb probe/Cy5 probe are shown. The fluorescence decay curves of all control measurements (without the presence of SH−SY5Y exosomes) in the donor and acceptor channel were normalized to be the same. Tb quenching and Cy5 sensitization in the presence of SH−SY5Y exosomes compared to that of control measurements demonstrated the FRET from Tb to Cy5. Also shown are the effects of the RCA duration (**C**), PLA probes (**D**), and backbone/connector (**E**) on FRET ratios. All measurements were performed with SH−SY5Y exosomes (20 ng∙μL^−1^), Cho primer/Apt−CD63 (10 nM), backbone/connector (10 nM), and Tb probe/Cy5 probe (20 nM) and the RCA duration time was set to 2 h, except in the special conditions indicated in the figures.

To enhance the sensitivity and the sensing performance of the APPROACH strategy, we optimized the key parameters in PLA, RCA, and TR-FRET. We initially optimized the concentrations of the TR-FRET probes (Tb probe and Cy5 probe) for detecting the RCA products by varying their concentrations from 20 nM to 300 nM. Decay curves were measured on a fluorescence spectrometer with a microplate reader equipped with a nitrogen laser source. Figure 4A,B show the normalized fluorescence decay curves of the donor and acceptor channels. Compared to control groups without the presence of exosomes, the PL intensities of Tb were quenched, while those of Cy5 were sensitized in all experimental settings, and new decay could be observed in both the Tb and Cy5 channels. This demonstrated the successful achievement of RCA products, as the quenched Tb and sensitized Cy5 signals were contributed to FRET from Tb to Cy5 probes which hybridized in proximity to RCA products. At relatively lower concentrations of TR-FRET probes, the majority of them hybridized to the RCA products, but as their concentrations continued to increase, the accessible hybridization sites of RCA products became exhausted, delimiting the proportion of probes that could hybridize to the RCA products and contribute to FRET signals; that is the reason why, with increasing concentrations of TR-FRET probes, normalized Tb quenching and Cy5 sensitization decreased in Figure 4A,B. Additionally, 20 nM and 50 nM of TR-FRET probes exhibited similar FRET sensitizations, and 50 nM was finally selected to balance reagent consumption and the quantitative measurement range.

The FRET ratios of the RCA process were continuously monitored from the starting point to 5 h and, as is shown in Figure 4C, the FRET ratios increased rapidly until 1 h and then slightly decreased, possibly due to the exhaustion of dNTPs or the prevention of TR-FRET probes from freely accessing the longer RCA products.

During the process of PLA, the concentrations of Cho primer, Apt-CD63, and backbone or connector play an important role in the formation of quadruplets, and thus, we varied the concentrations of Cho primer and Apt-CD63 simultaneously in the range of 0 nM to 50 nM. As depicted in Figure 4D, the FRET ratios increased gradually with the addition of PLA probes until 10 nM. The lower concentration of PLA probes might contribute to the limited amount of Cho primer inserted in proximity to CD63, thereby reducing the efficiency of PLA. Conversely, an excessive abundance of PLA probes would lead to the formation of triplets (Cho primer/Apt-CD63-backbone-connector) or duplets (Cho primer/Apt-CD63-backbone/connector), diminishing the quantity of the complete quadruplets necessary for the formation of circular DNA. Similarly, the concentrations of backbone and connector were varied simultaneously from 0 nM to 100 nM to test their effects on the formation of quadruplets. As shown in Figure 4E, the increasing concentrations of backbone and connector facilitated the formation of quadruplets; however, as the concentrations continued to increase after 20 nM, the excessive backbone and connector would prefer to form triplets or duplets instead of quadruplets, and consequently the FRET ratios started to decrease. A proper adjustment of the concentrations of PLA probes, backbone/connector, and TR-FRET probes was necessary for improving the performance of the ARRPOACH strategy, and the optimized parameters (10 nM for PLA probes, 20 nM for backbone/connector, and 50 nM for TR-FRET probes) were chosen for the following quantification of exosomal biomarkers.

### 3.5. Analytical Performance of Exosomal Biomarkers

**Figure 5:**
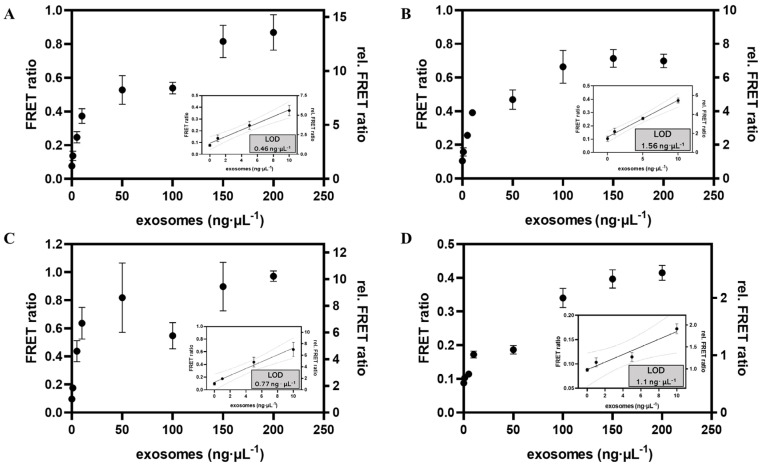
The APPROACH strategy calibration curves for three exosomal biomarkers. Shown are the calibration curves for SH−SY5Y exosomal CD63 in buffer (**A**) and in FBS (**B**), as well as A549 exosomal PD−L1 (**C**) and HER2 (**D**) in buffer. The final amounts or concentrations of other components in the 25 μL sample were: 10 nM PLA probes; 20 nM backbone/connector; 5 U of T4 DNA ligase; 5 U of phi29 DNA polymerase; 0.3 mM dNTP; and 50 nM TR−FRET probes. Data are represented as the means ± SDs (*n* = 3). LOD was determined as the concentration corresponding to the FRET ratio of the assay without any exosomes, plus 3 times standard deviation (3σ).

To evaluate the sensitivity of the proposed APPROACH strategy, we initially analyzed exosomal CD63 (derived from SH-SY5Y cells), which is widely expressed on the surface of exosomes [46]. The measurements were performed on a multifunctional microplate reader equipped with time-gated mode in black 384-well microtiter plates with a sample volume of 20 μL∙well^−1^. The samples contained constant concentrations of Cho primer and Apt-CD63 (10 nM), backbone and connector (20 nM), T4 DNA ligase (5 U), phi29 DNA polymerase (5 U), dNTPs (0.3 mM), and Tb and Cy5 probes (50 nM), as well as different concentrations of exosomes (ranging from 0 ng∙μL^−1^ to 200 ng∙μL^−1^) enriched with a certain amount of microspheres. As shown in Figure 5A, the APPROACH strategy showed a near-linear FRET ratio response of CD63 until 10 ng∙μL^−1^ of exosomes and then the FRET ratios started to saturate, possibly because of the exhaustion of at least one of the sensing reagents, such as microspheres, PLA probes, and TR-FRET probes; or a “huddle” of exosomes with RCA products on the surface of microsphere that prevented further amplification. The limit of detection (LOD) was calculated to be 0.46 ng∙μL^−1^ of CD63. The LOD was determined as the concentration corresponding to the signal intensity of the assay without any exosomes, plus three times standard deviation (3σ). Motivated by the remarkable sensitivity exhibited by APPROACH in detecting CD63 within the buffer, we proceeded to broaden the scope of our inquiry by evaluating the efficacy of the strategy in quantifying exosomal CD63 in fetal bovine serum (FBS). As shown in Figure 5B, the FRET ratio measured in FBS exhibited a similar response to that in buffer and the corresponding LOD was determined to be 1.56 ng∙μL^−1^. The APPROACH strategy employing TR-FRET demonstrated robust resistance to interference within complex serum matrices. Nevertheless, the sensitivity of the APPROACH strategy was slightly diminished when applied to serum samples, which might be attributed to the association of microspheres with serum proteins, which hindered exosomal enrichment, or the following coupling of PLA probes. 

Given the excellent sensing capability of the APPROACH strategy across various media, we further tested its applicability to other exosomal biomarkers. PD-L1 is vital in immune response regulation due to its interaction with its receptor PD-1 [47]. Exosomal PD-L1 serves as a pivotal mediator in immune evasion by obstructing T-cell activity and facilitating tumor immune escape. HER2 is overexpressed in cancers like breast cancer and lung cancer, and is associated with tumor development and progression [48]. Quantitative analysis of exosomal PD-L1 and HER2 contributes to biomarkers for tumor diagnosis and prognosis [25,49]. As illustrated in Figure 5C,D, the LODs for exosomal PD-L1 and HER2 (derived from A549 cells) were 0.77 ng∙μL^−1^ and 1.1 ng∙μL^−1^, respectively, comparable to that of exosomal CD63. The APPROACH strategy exhibited outstanding versatility, demonstrating its ability to effectively analyze various exosomal biomarkers.

The performance of the APPROACH strategy was then compared with several previously developed strategies, including fluorescent and electrochemical methods, surface plasmon resonance (SPR), surface-enhanced Raman spectroscopy (SERS), etc. (Table 1). These methods require either complex sample preparation procedures, intricate operational protocols, or specialized instrumentation, which limits their clinical practicality. The APPROACH strategy, by contrast, could be performed using different commercially available fluorescence plate reader systems found in most clinical testing centers, and it demonstrates competitive sensitivity with simplified analytical workflows, lower operational and technical demands, and improved applicability for complex biological matrices. These characteristics confer the advantages of the APPROACH strategy for in vitro diagnosis of exosomal biomarkers relative to other methods. Notably, the analytical performances of these strategies cannot be precisely compared due to the lack of a unified exosome quantification standard. Currently, both total protein amount per μL (ng∙μL^−1^) and particle number per μL (particles∙μL^−1^) are used to quantify exosomes. The dissimilarity arises from the variability of exosome sizes, and the fact that particle number is not directly proportional to total protein concentration. Despite these variations, the quantification of exosomes should be standardized in future investigations in order to fully compare the sensitivities and LODs of different emerging techniques, which could accelerate the process of broadening the application of exosome-based diagnostics in daily clinical practice.

## 4. Conclusions

We successfully developed and validated an APPROACH strategy for the quantitative detection of multiple exosomal biomarkers, demonstrating its wide applicability and ability in complex biological matrices. The semi-homogeneous detecting scenario due to the utilization of microspheres was a compromise between the interference of free PLA probes and minimal separation steps. In comparison to exosome analytical methods based on single recognition elements, the APPROACH strategy employs an oligonucleotide coupled with cholesterol and PLA to enhance its specificity in detecting exosomal biomarkers. The enhanced specificity stems from the simultaneous recognition of the biolipid layer and exosomal biomarkers, effectively eliminating potential non-specific fluorescence signals commonly associated with free proteins. Compared with the methods employing antibodies, aptamers can efficiently convert protein signals into nucleic acid signals with no requirement of intricate coupling processes, achieving signal amplification through isothermal amplification techniques. Moreover, unlike methods based on Raman and mass spectrometry, the APPROACH strategy utilizes microspheres and TR-FRET to facilitate semi-homogeneous detection without the need for expensive equipment, and the cost-effectiveness and simplified workflow make it more favorable for early-stage diagnosis and personalized treatment. As an ideal situation for exosome detection would be totally homogeneous, developing a sensing strategy without the assistance of microspheres or other separation steps should be highlighted in future investigations. Furthermore, by employing FRET acceptors featuring distinct fluorescence colors and/or positioned at controlled distances from the lanthanide FRET donor, the APPROACH strategy has the potential to access to both spectral (color) and temporal (lifetime) PL multiplexing, which can be integrated for higher-order multiplexed and quantitative exosomal biomarkers. We believe the strategy holds great promise for applications such as in vitro diagnostic research of exosomes.

## Figures and Tables

**Table 1:** Brief comparison of various exosome identification strategies.

Strategy	Identification Target	Linear Range	LOD	Reference
Size-coded affinity microbeads	PD-L1, EpCAM, EGFR	0–60 ng∙μL^−1^	---	[25]
Electrochemical sandwich immunosensor	α-CD9	10^2^–10^6^ particles∙μL^−1^	200 particles∙μL^−1^	[50]
Fluorescence detection	CD63	10^3^–10^7^ particles∙μL^−1^	---	[6]
Total membrane lipid assay	Bilipid layer	0–200 ng∙μL^−1^	0.342 ng μL^−1^	[51]
DNAzyme- based method	PD-L1	0–1000 ng∙μL^−1^	5.21 ng∙μL^−1^	[52]
Magnetic-based microfluidic device	CD63	7.6 × (10^1^–10^5^) particles ∙μL^−1^	4.39 particles∙μL^−1^	[53]
SERS-based method	MUC1, HER2, CEA	1–3 × 10^7^ particles∙mL^−1^	10^7^–10^12^ particles∙mL^−1^	[49]
SPR-based method	PD-L1	10–5000 particles ∙μL^−1^	0.0167 particles∙μL^−1^	[54]
CRISPR/Cas12a	CD63	3 × 10^3^–6 ×10^7^ particles∙μL^−1^	---	[55]
APPROACH	CD63, PD-L1, HER2	0–200 ng∙μL^−1^	0.46 ng∙μL^−1^, 0.77 ng∙μL^−1^, 1.1 ng∙μL^−1^	This work

---: not mentioned.

## Data Availability

Data are contained within the article and Supplementary Materials.

## Supplementary Materials

The following supporting information can be downloaded at: https://www.mdpi.com/article/10.3390/bios14050233/s1. Table S1: Sequences of oligonucleotides used in this work; Figure S1: Spectral characterization of fluorescent probes; Figure S2: Flow cytometry analysis of RCA products (RCPs); Figure S3: The HRMS spectrum of [CoraFluor-like-NHS].
