# Supplementary material for: APPROACH: Sensitive Detection of Exosomal Biomarkers by Aptamer-Mediated Proximity Ligation Assay and Time-Resolved Förster Resonance Energy Transfer
Source: Biosensors (Basel). 2024 May 8;14(5):233. doi: 10.3390/bios14050233 (PMC11117858; doi:10.3390/bios14050233)
Supplement: Supplementary file 1 [file biosensors-14-00233-s001.zip › biosensors-2972666-supplementary.pdf]

APPROACH: Sensitive Detection of Exosomal Biomarkers by Aptamer-Mediated Proximity Ligation Assay and Time-Resolved Förster Resonance Energy Transfer

Ying Li [1,†], Meiqi Qian [1,†], Yongpeng Liu [2] and Xue Qiu [1,*]

1 Key Laboratory of Marine Drug, Ministry of Education, School of Medicine and Pharmacy, Ocean University of China, Qingdao 266003, China; Laboratory for Marine Drugs and Bioproducts, Qingdao National Laboratory for Marine Science and Technology, Qingdao 266237, China; liying9239@stu.ouc.edu.cn (Y.L.); qmq@stu.ouc.edu.cn (M.Q.)

2 BGI Research, Shenzhen 518083, China; liuyongpeng@genomics.cn

* Correspondence: qiuxue@ouc.edu.cn

† These authors contributed equally to this work.

Table S1. Sequences of oligonucleotides used in this work……………………………………2
Figure S1. Spectral characterization of fluorescent probes……………………………………3
Figure S2. Flow cytometry analysis of RCA products (RCPs)…………………………………3
Figure S3. The HRMS spectrum of [CoraFluor−like−NHS]……………………………………4

Table S1. Sequences of oligonucleotides used in this work.

Name	Sequence (5′-3′)
Apt-CD63[1]	ATATACACCCCACCTCGCTCCCGTGACACTAATGCTATTTTTTTTTT TTTTTGACGCTAATAGTTAAGACGCTT
Cho primer[1]	Cholesterol-GACCCTAAGCATACATGCTCACTGACGCTAGGTTTTTTTTTT TTTTATATGACAGAACTAGACACTCTT
Apt-HER2	TCTAAAAGGATTCTTCCCAAGGGGATCCAATTCAAACAGCTTTTT TTTTTTTTTTGACGCTAATAGTTAAGACGCT
Apt-PD-L1	ACGGGCCACATCAACTCATTGATAGACAATGCGTCCACTGCCCG TTTTTTTTTTTTTTTGACGCTAATAGTTAAGACGCT
Backbone[1]	Phosphate-CTATTAGCGTCCAGTGAATTATACCCGGTCGCTTCTTTATGCC GTCAAGAGTGTCTA
Connector[1]	Phosphate-GTTCTGTCATATTTAAGCGTCTTAA
Tb probe	Tb-TGAATTATACCCGGT (conjugation ratio: 0.87 Tb per DNA)
Cy5 probe	Cy5-CGCTTCTTTATGCCG (conjugation ratio: 0.92 Cy5 per DNA)
Cho primer-Cy5	Cholesterol-.GACCCTAAGCATACATGCTCACTGACGCTAGGTTTTT TTTTTTTTTTATATGACAGAACTAGACACTCTT-Cy5 (conjugation ratio: 1.5 Cy5 per DNA)
Apt-CD63-Cy5	ATATACACCCCACCTCGCTCCCGTGACACTAATGCTATTTTTTTTTT TTTTTGACGCTAATAGTTAAGACGCT-Cy5 (conjugation ratio: 1.5 Cy5 per DNA)
MU-Cy5	Cy5-CTTTTCGGGATTTATGC (conjugation ratio: 1 Cy5 per DNA)

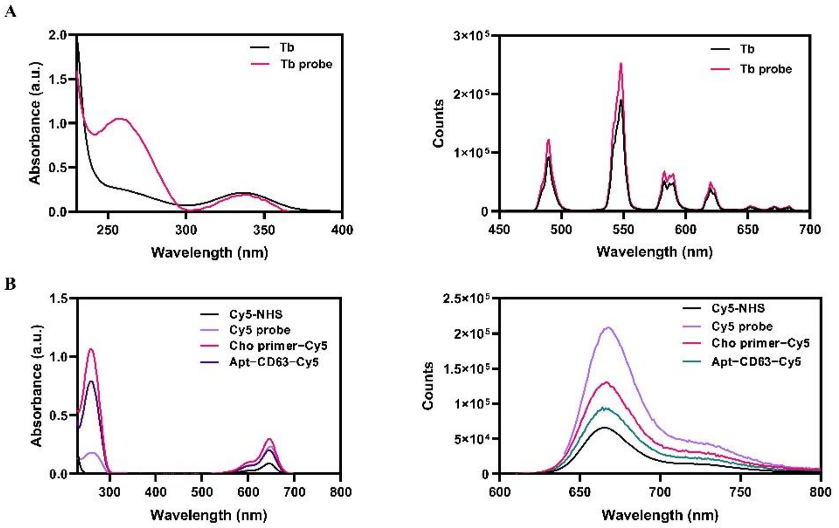

Figure S1. Spectral characterization of Tb probe, Cy5 probe, Cho priemr−Cy5 and Apt−CD63−Cy5. The concentrations and labeling ratios (dye/oligonucleotides) were determined through absorption spectroscopy. The absorbance and emission spectra of Tb and Cy5 probes were presented in (A) and (B), respectively.

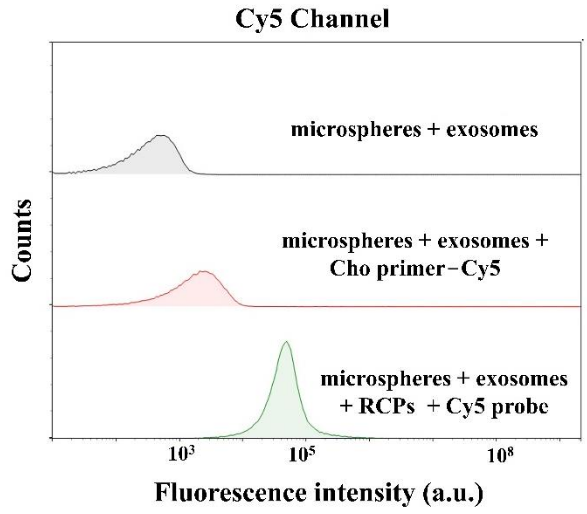

Figure S2. Flow cytometry analysis of RCA products (RCPs).

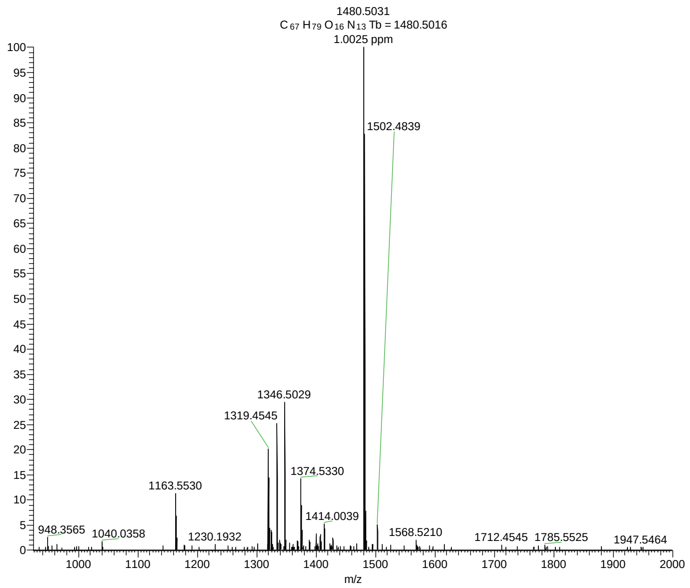

Figure S3. The HRMS spectrum of [CoraFluor−like−NHS].

References:

1. Zhao, X.; Luo, C.; Mei, Q.; Zhang, H.; Zhang, W.; Su, D.; Fu, W.; Luo, Y., Aptamer-Cholesterol-Mediated Proximity Ligation Assay for Accurate Identification of Exosomes. *Anal. Chem.* 2020, *92* (7), 5411-5418, DOI:/10.1021/acs.analchem.0c00141.
